# Negative life events and mobile phone addiction among Chinese vocational college students: a chain-mediation model of perceived stress and psychological resilience

**DOI:** 10.3389/fpsyg.2026.1854552

**Published:** 2026-06-17

**Authors:** Lijuan Xu, Li Li

**Affiliations:** 1 School of Education, Jiangxi Institute of Applied Science and Technology, Nanchang, China; 2 Faculty of Psychology, Nanchang University, Nanchang, China

**Keywords:** Chinese vocational college students, mobile phone addiction, negative life events, perceived stress, psychological resilience

## Abstract

**Background:**

Mobile phone addiction not only poses potential adverse effects on the effective implementation of educational and teaching activities, but also exerts a detrimental influence on students’ mental health. Negative life events have been identified as a significant contributing factor to mobile phone addiction among vocational college students. Although prior research has investigated the relationship between life events and problematic mobile phone use, this study represents the first comprehensive examination of the sequential mediating roles of perceived stress and psychological resilience in the association between negative life events and mobile phone addiction. This investigation not only extends the application of stress-coping theory within the domain of digital behavior addiction, but also offers novel empirical insights and potential intervention strategies for understanding the psychological vulnerability and adaptive mechanisms involved in the development of mobile phone addiction among vocational college students.

**Objective:**

The present study investigates the relationship between perceived stress and psychological resilience in the context of negative life events and mobile phone addiction, as well as the underlying mechanisms that mediate this association.

**Methods:**

Data collection was carried out among 587 vocational college students in Nanchang City, Jiujiang City, and Fuzhou City of Jiangxi Province, using the Self-Rated Life Events Scale for Adolescents, the Perceived Stress Scale, the Psychological Resilience Scale, and the Mobile Phone Addiction Questionnaire.

**Results:**

The findings are: (1) Mobile phone addiction is widespread among vocational college students, and the level of mobile phone addiction does not show significant differences in factors such as gender and place of origin. (2) Negative life events among vocational college students are positively associated with mobile phone addiction. (3) Perceived stress and psychological resilience play an independent mediating role and a chain mediating role between negative life events experienced by vocational college students and mobile phone addiction behaviors. The mediating effect includes three paths: negative life events → perceived stress → mobile phone addiction (effect size: 0.200), negative life events → psychological resilience → mobile phone addiction (effect size: 0.047), and negative life events → perceived stress → psychological resilience → mobile phone addiction (effect size: 0.024).

**Conclusion:**

This study highlights that negative life events are directly associated with mobile phone addiction among vocational college students, and also are indirectly associated with the degree of mobile phone addiction among vocational college students through the mediating effects of perceived stress and psychological resilience. The research results provide an important theoretical basis for higher vocational colleges to deeply understand the psychological causes of students’ mobile phone addiction, which is conducive to optimizing the content of mental health education in schools and strengthening stress management training and psychological resilience cultivation in a targeted manner. It provides scientific support for educators to design stratified intervention plans and prevent problematic mobile phone usage behaviors, and also offers a powerful practical reference for promoting the formulation of digital health promotion policies for vocational college students and creating a positive and healthy campus network environment.

## 1 Introduction

The high-quality development of vocational education serves as a crucial driving force behind China’s modernization efforts, with the all-round development of students, particularly their physical and mental well-being, representing a key component of this process. However, mobile phone addiction poses a serious threat to the mental health of vocational college students. Mobile phone addiction refers to an individual’s strong dependence on and persistent psychological craving for mobile phone–related activities (Liu et al., 2017). Excessive engagement in such behavior may negatively affect physical health, mental well-being, social adaptability, and academic performance. Research indicates that prolonged mobile phone addiction is linked to a range of health issues, including vision impairment, cardiovascular diseases, obesity, fatigue, cognitive decline, hearing loss, and sleep disturbances (Demirci et al., 2015; Mason et al., 2022; Zhang B. et al., 2019; Zhang M. et al., 2019; Zhang X. et al., 2019; Vacaru et al., 2014). Meanwhile, research shows that mobile phone addiction may also trigger several mental health problems, such as feelings of loneliness (Mei et al., 2023), anxiety (Lin et al., 2023), and depression (Wang and Sun, 2022), which may further lead to social anxiety (Darcin et al., 2016; Zeng et al., 2025) and tense interpersonal relationships (Liu et al., 2020). It can also weaken an individual’s level of learning commitment (Wei, 2023), increase the risk of academic burnout (Zhang B. et al., 2019; Zhang M. et al., 2019; Zhang X. et al., 2019), and ultimately have an adverse impact on academic performance. The 55th “Statistical Report on the Development of China’s Internet” released by the China Internet Society shows that as of December 2024, the number of people accessing the Internet via mobile phones in China exceeded 1.105 billion, an increase of 14.03 million compared with the same period of the previous year. The proportion of Internet users accessing the Internet via mobile phones reached 99.7%, almost covering the entire Internet user group. The problem of mobile phone addiction among vocational college students is serious. Existing studies have shown that a considerable number of vocational college students display varying degrees of mobile phone addiction tendencies (Liu et al., 2024). Wang Jing conducted a follow-up study on 361 vocational college students and found that the level of mobile phone addiction showed a continuous upward trend (T1: 36.22 ± 11.89; T2: 37.19 ± 13.01; T3: 39.07 ± 14.22) (Wang, 2024). These findings indicate that mobile phone addiction among vocational college students has become an important educational and mental health issue that urgently requires attention and intervention.

### 1.1 Negative life events and mobile phone addiction

Negative life events refer to adverse experiences that individuals have encountered in the recent or distant past, often accompanied by negative emotional responses such as anxiety, depression, and distress (He, 2008). Prior research has demonstrated that life events, as critical components of the social environment, exert a significant influence on addictive behaviors, including substance dependence and Internet addiction (Charles et al., 2013). Furthermore, a substantial body of cross-sectional evidence indicates that the greater the number of negative life events an individual experiences, the higher their level of mobile phone dependence, which contributes to increased addictive behaviors (Wan, 2020; Wang, 2021). When individuals encounter adverse life events that elevate stress levels, they may turn to mobile phones as a means of emotional relief. The pleasure and temporary alleviation of tension experienced during usage can reinforce the behavior through positive reinforcement mechanisms, increasing the likelihood of frequent use and ultimately the risk of developing mobile phone addiction. Davis’s cognitive-behavioral model offers a comprehensive explanation of the internal mechanisms underlying mobile phone addiction and elucidates the dynamic progression of this phenomenon (Tian et al., 2023; Wu, 2022). This theoretical framework highlights that mobile phone addiction is not only driven by proximal factors (such as maladaptive cognition and inadequate social support systems) but is also indirectly influenced by distal psychopathological factors (such as depression and anxiety). These distal factors amplify an individual’s vulnerability to mobile phone addiction by modulating proximal triggers (Davis, 2001). Within the context of this model, negative life events can be conceptualized as a distal factor that exerts a direct influence on mobile phone addiction behaviors. Therefore, the current study proposes the following:

*H1*: Negative life events has a significant positive predictive effect on Chinese vocational college students’ mobile phone addiction.

### 1.2 The mediating role of perceived stress

Although Hypothesis 1 emphasizes the direct predictive relationship between negative life events and mobile phone addiction, cognitive-behavioral models propose that this distal factor may elevate an individual’s perceived stress levels, potentially leading to maladaptive cognitive patterns and increased reliance on mobile phones (Davis, 2001). According to Cohen et al. (1983), “perceived stress” refers to an individual’s subjective psychological response when encountering various stressors, particularly when these events are perceived as challenging or threatening. As a prevalent source of stress, negative life events often trigger negative cognitive appraisals, intensifying individuals’ subjective experience of stress. Empirical evidence further supports this assertion, indicating that adolescents who experience a greater number of negative life events tend to exhibit higher levels of perceived stress (Li et al., 2022). Building on McEwen’s theory of adaptation and the comprehensive stress process model, it becomes evident that multiple interrelated factors, including external stressors, individual cognitive appraisals, neuroendocrine responses, and overall psychological and physical health, interact within the stress response system (Ganzel et al., 2010; McEwen, 2003). This model suggests that prolonged exposure to stressful environments, coupled with sustained activation of physiological systems, may hinder an individual’s capacity for effective adaptation, potentially resulting in various psychological and behavioral issues such as depression and mobile phone addiction. Empirical research indicates that an individual’s perceived stress level can significantly and positively predict the likelihood of mobile phone addiction. In other words, higher stress levels are associated with a greater tendency to use mobile phones frequently as a means of coping with negative emotions, which increases the risk of developing an addiction (Lian et al., 2018; Xu et al., 2024). Therefore, based on the above theoretical framework and empirical findings, this study considers perceived stress to be a mediating variable and proposes the following:

*H2*: Perceived stress plays a mediating role in the relationship between negative life events and mobile phone addiction among Chinese vocational college students.

### 1.3 The mediating role of psychological resilience

As a critical psychological construct, psychological resilience may function as an important mediating factor in the association between negative life events and mobile phone addiction. Psychological resilience is defined as an individual’s capacity to adapt effectively and recover promptly when confronted with adversity or psychological stress (Connor and Davidson, 2003). Although substantial research has established that psychological resilience can buffer the detrimental effects of stressful experiences (Sheerin et al., 2018), sustained exposure to high-pressure environments may deplete an individual’s internal psychological and emotional regulatory resources, reducing their ability to manage stress effectively. Empirical studies have indicated that individuals who encounter a greater frequency of negative life events are more likely to exhibit diminished levels of psychological resilience (Chen et al., 2017). As a result, individuals with lower resilience may increasingly depend on external coping strategies, such as mobile phone usage, to regulate emotional distress and avoid real-life stressors, which may subsequently elevate the risk of developing addictive behaviors. According to the Resilience Framework theory, psychological resilience is not a fixed personality trait but a dynamic and multidimensional adaptive process. Individuals with higher resilience generally exhibit fewer maladaptive behavioral outcomes (such as depression, anxiety, and addictive tendencies) and demonstrate better maintenance of social and functional capabilities (Kumpfer and Bluth, 2004). Therefore, reduced levels of psychological resilience may substantially elevate susceptibility to mobile phone dependency. Yu (2016) research further corroborates this perspective, demonstrating a statistically significant negative correlation between overall mobile phone dependence levels and psychological resilience, extending to its respective subdimensions. Drawing upon the above theoretical and empirical evidence, we propose the following:

*H3*: Psychological resilience acts as an intermediary mechanism in the relationship between negative life events and mobile phone addiction.

### 1.4 The chain-mediating role of perceived stress and psychological resilience

Research on the psychological mechanisms underlying the relationship between stress response and adaptive behavior has increasingly focused on the interaction between perceived stress and psychological resilience. The level of perceived stress in individuals may serve as a critical psychological determinant of their resilience. Extant studies have consistently shown that perceived stress negatively predicts psychological resilience (Ye et al., 2018), suggesting that heightened levels of perceived stress directly impair emotional regulation and reduce the availability of psychological resources, thereby undermining individuals’ ability to manage adversity and regulate emotions effectively. Moreover, empirical evidence indicates a significant sequential mediating role of perceived stress and psychological resilience in relation to nonrestorative sleep among college freshmen (Liao et al., 2023a,b). Based on these findings, this study proposes that negative life events may increase the likelihood of mobile phone addiction among vocational college students by initiating a psychological pathway involving elevated perceived stress and subsequent reductions in psychological resilience. In this context, perceived stress and psychological resilience operate as sequential mediators. Consequently, we propose the following:

*H4*: There is a chain-mediation effect between perceived stress and psychological resilience in the relationship between negative life events and mobile phone addiction.

In conclusion, grounded in both theoretical frameworks and empirical evidence, this study develops a sequential mediation model to examine systematically the influence of negative life events on mobile phone addiction among vocational college students. It further elucidates the mediating roles of perceived stress and psychological resilience in this relationship. These findings offer a theoretical foundation and practical insights for promoting mental health, improving academic adaptability, and refining educational management strategies.

## 2 Materials and methods

### 2.1 Participants

This study conducted a five-week survey among students from four vocational colleges in Nanchang City, Jiujiang City, and other locations in Jiangxi Province between May 28 and July 2, 2025, utilizing a convenience sampling method. A total of 643 questionnaires were distributed through online and paper formats, and 587 valid responses were collected, resulting in an effective response rate of 91.30%. The sample included 247 students (42.08%) from Grade One, 197 students (33.56%) from Grade Two, and 143 students (24.36%) from Grade Three. The gender distribution comprised 235 male students (40.03%) and 352 female students (59.97%). Regarding regional background, 353 participants were from urban areas (60.14%) and 234 were from rural areas (39.86%). All participants voluntarily joined the survey and signed informed consent forms. The study was approved by the Ethics Committee of Nanchang University. Although the relatively large sample size (*N* = 587) contributes to more stable parameter estimation and reduced sampling error, the use of convenience sampling and reliance on a single-source dataset drawn exclusively from one province limit the representativeness of the sample for the national population of vocational college students.

### 2.2 Measurements

#### 2.2.1 Negative life events

The Adolescent Life Events Self-Rating Scale (Liu et al., 1997) was employed to evaluate the stress-related experiences of students over the past 12 months. The scale encompasses six dimensions: interpersonal relationships, academic pressure, punishment, loss, health, and adaptation, comprising 27 items. A five-point Likert scale ranging from 1 to 5 was used, where 1 indicates no occurrence or impact and 5 indicates an extremely significant impact, with higher scores indicating a greater impact of events. Although originally developed for adolescents, this scale has been validated in prior studies and is widely employed among Chinese college and vocational students (Hu et al., 2011; Chen et al., 2020; He et al., 2023). The internal consistency of the scale in this study was excellent, as evidenced by a Cronbach’s alpha coefficient of 0.964. Among these, 300 data points were selected to conduct a confirmatory factor analysis on the questionnaire, resulting in an acceptable fit of the questionnaire structure (χ^2^/df = 3.840, CFI = 0.852, TLI = 0.832, and IFI = 0.853).

#### 2.2.2 Perceived stress

The Perceived Stress Scale (Yang and Huang, 2003) was revised by Professor Yang and Huang based on Chinese cultural contexts. This revised version comprises 14 items, categorized into two dimensions: perceived tension and sense of loss of control. Specifically, items 4, 5, 6, 7, 9, 10, and 13 are reverse-scored and correspond to the dimension of loss of control, whereas the remaining items are positively scored and pertain to the dimension of tension. A five-point Likert-type scoring system is employed, with higher total scores indicating greater perceived stress levels. This scale has been previously validated and widely applied in Chinese college and vocational student populations (Ye et al., 2018; Jia et al., 2022; Wang and Wang, 2023). The scale demonstrates strong internal consistency, as evidenced by a Cronbach’s alpha coefficient of 0.877, reflecting its reliability and validity. Among these, 300 data points were selected to conduct a confirmatory factor analysis on the questionnaire, resulting in an acceptable fit of the questionnaire structure (χ^2^/df = 2.887, CFI = 0.940, TLI = 0.929, and IFI = 0.941).

#### 2.2.3 Psychological resilience

The psychological resilience scale was initially developed by Ye et al. through simplification and optimization of a 25-item psychological resilience scale. Subsequently, domestic scholars Ye et al. (2016) completed the translation and cultural adaptation of the Chinese version. The scale exhibits a unidimensional structure and comprises 10 items, such as “I can adapt when things change” and “I can recover easily from illness, injury, or difficulty.” A five-point Likert scoring method is employed. The total score serves as a key indicator for measuring an individual’s positive psychological capital, with higher scores reflecting stronger stress tolerance and adaptability. This scale has been previously validated and widely applied in Chinese college and vocational student populations (Liao et al., 2023a,b; Ye et al., 2016). Reliability analysis revealed a Cronbach’s alpha coefficient of 0.937 for the overall scale, indicating satisfactory internal consistency in this study. Among these, 300 data points were selected to conduct a confirmatory factor analysis on the questionnaire, resulting in an acceptable fit of the questionnaire structure (χ^2^ /df = 2.449, CFI = 0.975, TLI = 0.967, and IFI = 0.975).

#### 2.2.4 Mobile phone addiction

The term “mobile phone addiction” has been widely adopted in Chinese psychological literature to denote patterns of excessive and clinically significant mobile phone use (Xiong et al., 2012). However, it is important to note that this construct remains conceptually contested in the international scholarly literature. In alignment with the more circumspect terminological conventions prevailing in global academic discourse, the present study operationalizes the phenomenon as “problematic mobile phone use”—a behavioral pattern characterized by excessive, maladaptive, or functionally impairing engagement with mobile devices—rather than as a formal clinical diagnosis of behavioral addiction.

The Mobile Phone Addiction Questionnaire was developed by Xiong et al. (2012) and comprises four dimensions: withdrawal symptoms (items 1, 4, 6, 8, 10, 12), salient behaviors (items 5, 9, 13, 15), social comfort (items 2, 7, 16), and mood changes (items 3, 11, 14). The questionnaire does not include any reverse-scored items. It employs a five-point Likert scale ranging from 1 (completely inconsistent) to 5 (very consistent). The total score is positively correlated with the severity of mobile phone addiction, with higher scores indicating more pronounced addiction. This scale has been previously validated and widely applied in Chinese college and vocational student populations (Zhang B. et al., 2019; Zhang M. et al., 2019; Zhang X. et al., 2019; Wang, 2019; Li et al., 2021). In this sample, the Cronbach’s alpha coefficient for the entire scale was 0.922, suggesting a high level of internal consistency and reliability. Among these, 300 data points were selected to conduct a confirmatory factor analysis on the questionnaire, resulting in an acceptable fit of the questionnaire structure (χ^2^/df = 3.284, CFI = 0.906, TLI = 0.885, and IFI = 0.907).

### 2.3 Data analysis

The data were statistically analyzed using SPSS 27.0 and PROCESS 4.2. Common method bias was assessed using Harman’s single-factor test, and procedural remedies (anonymous responding, psychologically separated instructions, reverse-coded items, and randomized section order) were implemented to minimize its potential impact. Descriptive statistics were then employed, and the reliability of the scales was evaluated using Cronbach’s alpha. Pearson correlation coefficients were calculated to explore variable relationships. Academic grade was coded as 1, 2, and 3 for Grade One, Grade Two, and Grade Three, respectively, and was included as a covariate in the subsequent mediation analysis because students at different stages face systematically different academic loads, social environments, and developmental tasks. Finally, PROCESS (Model 6) was used to examine chain-mediation relationships involving negative life events, perceived stress, psychological resilience, and mobile phone addiction.

## 3 Results

### 3.1 Results of the common method biases test

The use of self-reported questionnaires in this study may introduce common method bias, potentially affecting the accuracy of the results (Zhou and Long, 2004). To assess this concern, a Harman’s one-factor test was performed on all items from the four measurement instruments. Exploratory factor analysis indicated that, without rotation, 10 factors had eigenvalues greater than 1. The first factor accounted for 33.05% of the total variance, which is below the critical threshold of 40%. However, the sensitivity of this test is inherently limited; thus, its results should be interpreted solely as indicative—rather than definitive—evidence regarding the absence of common method bias. To mitigate such bias, four procedural safeguards were implemented during data collection: (1) anonymous and voluntary survey completion, (2) psychological separation of scale instructions to minimize response carryover effects, (3) inclusion of reverse-scored items to counteract acquiescence bias, and (4) randomization of item order across questionnaire sections (Siemsen, 2010). Furthermore, the present study examines a complex theoretical model featuring multiple mediating pathways—a structure that is unlikely to be fully attributable to common method bias.

### 3.2 Descriptive statistics and correlation analyses of the research variables

This study examines four core variables: negative life events, perceived stress, psychological resilience, and mobile phone addiction. The descriptive statistical results and correlation analysis for each variable are presented in Table 1. The data show that negative life events exhibit a statistically significant positive association with perceived stress (*r* = 0.423, *p* < 0.001) and mobile phone addiction (*r* = 0.571, *p* < 0.001), while demonstrating a significant negative relationship with psychological resilience (*r* = −0.467, *p* < 0.001). Perceived stress is positively and significantly correlated with mobile phone addiction (*r* = 0.661, *p* < 0.001) and inversely related to psychological resilience (*r* = −0.508, *p* < 0.001). In addition, psychological resilience shows a significant negative correlation with mobile phone addiction (*r* = −0.523, *p* < 0.001). Together, these results highlight the interconnectedness among the studied variables, providing a solid empirical basis for hypothesis development and testing. The correlations provide a foundation for validating the subsequent hypotheses. The study further revealed significant correlations between academic grade level and negative life events (*r* = −0.456, *p* < 0.01), perceived stress (*r* = −0.516, *p* < 0.01), psychological resilience (*r* = 0.369, *p* < 0.01), and mobile phone addiction tendency (*r* = −0.713, *p* < 0.01). Subsequent one-way ANOVA with *post hoc* comparisons indicated statistically significant differences across grade levels for all four variables: higher-grade students reported lower frequencies of negative life events, lower levels of perceived stress, reduced mobile phone addiction tendency, and higher levels of psychological resilience. The results are presented in Table 2.

**Table 1:** Descriptive statistics and correlation matrix of each variable (*n* = 587).

Variable	M ± SD	1	2	3	4	5	6
1 Negative life events	1.798 ± 0.677	1					
2 Perceived stress	2.785 ± 0.655	0.423^***^	1				
3 Psychological resilience	3.192 ± 0.772	−0.467^***^	−0.508^***^	1			
4 Mobile phone addiction	2.582 ± 0.703	0.571^***^	0.661^***^	−0.523^***^	1		
5 Gender	—	0.007	0.073	−0.043	0.045	1	
6 Place of origin	—	0.063	0.053	−0.025	0.004	−0.016	1
7 Grade	—	−0.456^***^	−0.516^***^	0.369^***^	−0.713^***^	−0.051	0.015

^*^p < 0.05, ^**^p < 0.01, ^***^p < 0.001.

**Table 2:** Grade-based comparative analysis of study variables (*n* = 587).

Variable	①First year of vocational college	②Second year of vocational college	③Third year of vocational college	*F*	*p*	LSD
Negative life events	2.127 ± 0.719	1.704 ± 0.607	1.358 ± 0.302	77.085	0.000	①>②>③
Perceived stress	3.069 ± 0.527	2.871 ± 0.474	2.172 ± 0.669	124.282	0.000	①>②>③
Psychological resilience	2.896 ± 0.729	3.258 ± 0.663	3.611 ± 0.773	46.039	0.000	③>②>①
Mobile phone addiction	3.065 ± 0.508	2.554 ± 0.455	1.782 ± 0.501	311.469	0.000	①>②>③

*p < 0.05, **p < 0.01, ***p < 0.001.

### 3.3 Analysis of mediation effects

Given the cross-sectional nature of the data, the following results should be interpreted as indicating statistical indirect effects consistent with the proposed sequential model, rather than as evidence of a causal temporal sequence. A multiple regression model was constructed with negative life events, perceived stress, and psychological resilience as independent variables and mobile phone addiction as the dependent variable. The resulting variance inflation factor (VIF) values were all less than 5, indicating an absence of multicollinearity among the predictors. To further investigate the relationships among negative life events, perceived stress, psychological resilience, and mobile phone addiction, the SPSS PROCESS macro program (Model 6) was used. In this analysis, negative life events were treated as the independent variable and mobile phone addiction as the dependent variable. Perceived stress and psychological resilience were included as mediating variables, while gender, place of origin, and academic grade were controlled for as covariates. The analysis aimed to examine the sequential mediating effect of the two mediators.

**Figure 1:**
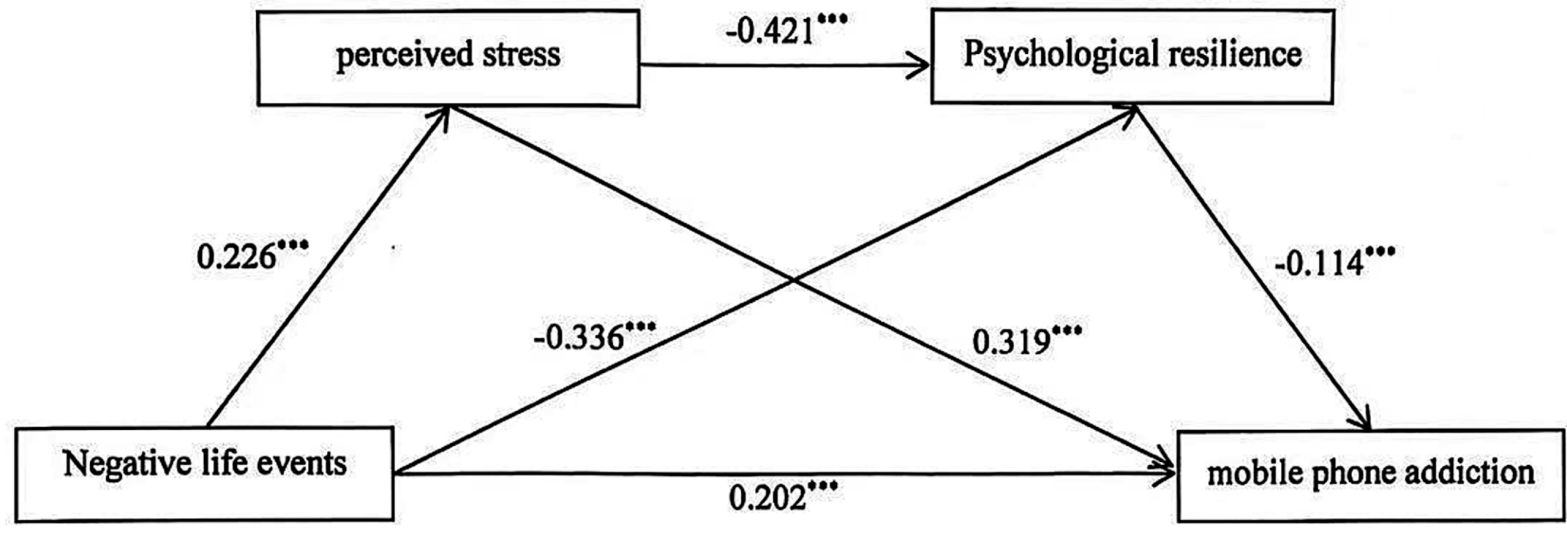
Serial mediation model (*p < 0.05, **p < 0.01, ***p < 0.001).

The results are presented in Table 3 and Figure 1. Negative life events showed significant positive associations with perceived stress (*β* = 0.226, *p* < 0.001) and significant negative associations with psychological resilience (*β* = −0.336, *p* < 0.001). In addition, perceived stress was significantly and negatively associated with psychological resilience (*β* = −0.421, *p* < 0.001) and significantly and positively associated with mobile phone addiction (*β* = 0.319, *p* < 0.001). Finally, both negative life events and psychological resilience demonstrated significant associations with mobile phone addiction (β₁ = 0.202, *p* < 0.001; β₂ = −0.114, *p* < 0.001). These findings suggest that perceived stress and psychological resilience exert both partial and chain-mediating effects between negative life events and mobile phone addiction, suggesting that these variables play multiple psychological roles in this relationship.

**Table 3:** Regression analysis of the research variables.

Variable	Perceived stress			Psychological resilience			Mobile phone addiction		
	β	SE	t	β	SE	t	β	SE	t
Gender	0.069	0.046	1.496	−0.020	0.053	−0.371	−0.008	0.034	−0.228
Place of origin	0.061	0.046	1.319	0.019	0.053	0.347	−0.030	0.034	−0.883
Grade	−0.335	0.032	−10.554^***^	0.048	0.040	1.189	−0.375	0.026	−14.586^***^
Negative life events	0.226	0.037	6.055^***^	−0.336	0.045	−7.498^***^	0.202	0.030	6.746^***^
Perceived stress				−0.421	0.048	−8.756^***^	0.319	0.033	9.759^***^
Psychological resilience							−0.114	0.026	−4.314^***^
*R* ^2^	0.316			0.338			0.675		
F	67.150^***^			59.209^***^			201.150^***^		

^*^p < 0.05, ^**^p < 0.01, ^***^p < 0.001.

Subsequently, the chain-mediation model was analyzed using the bootstrap method with 5,000 resamples and a 95% confidence interval. A mediation effect is considered statistically significant if the confidence interval does not include zero (Wen and Ye, 2014). The results are summarized in Table 4. The total effect of negative life events on mobile phone addiction was 0.592 (95% CI [0.523, 0.662]), which was statistically significant. The direct effect was 0.321 (95% CI [0.256, 0.387]), representing 54.22% of the total effect and also statistically significant, consistent with Hypothesis H1. The indirect effect was decomposed into three pathways: (1) negative life events → perceived stress → mobile phone addiction, with an effect size of 0.200 (95% CI [0.153, 0.251]), accounting for 33.78% of the total effect and consistent with Hypothesis H2; (2) negative life events → psychological resilience → mobile phone addiction, with an effect size of 0.047 (95% CI [0.022, 0.079]), representing 7.94% of the total effect and consistent with Hypothesis H3; (3) the serial mediating pathway of negative life events → perceived stress → psychological resilience → mobile phone addiction yielded an effect size of 0.024 (95% CI [0.012, 0.039]), accounting for 4.06% of the total effect and consistent with Hypothesis H4. All three indirect pathways were statistically significant, indicating that perceived stress and psychological resilience partially and sequentially account for indirect associations between negative life events and mobile phone addiction.

**Table 4:** The results of mediating effect test.

Model paths	Effect size	Boot SE	95% LLCI	95% ULCI	Ratio
Direct effect					
Negative life events → mobile phone addiction	0.321	0.034	0.256	0.387	54.22%
Indirect effect					
Path 1: negative life events→ perceived stress → mobile phone addiction	0.200	0.025	0.153	0.251	33.78%
Path 2: negative life events→ psychological resilience → mobile phone addiction	0.047	0.015	0.022	0.079	7.94%
Path 3: negative life events→ perceived stress→ psychological resilience → mobile phone addiction	0.024	0.007	0.012	0.039	4.06%
Total effect	0.592	0.035	0.523	0.662	

LLCI, lower limit of the confidence interval; SE, standard error; ULCI, upper limit of the confidence interval.

## 4 Discussion

This study examined the interrelationships among negative life events, perceived stress, psychological resilience, and mobile phone addiction in vocational college students. The findings show that negative life events are not only directly associated with mobile phone addiction but also indirectly linked to greater addictive behaviors through higher levels of perceived stress. Psychological resilience functions as a mediator alongside perceived stress in this process. The research sheds light on the underlying mechanisms associated with mobile phone addiction and provides a theoretical basis for intervention strategies. These results suggest that mitigating stress and fostering psychological resilience may be important for addressing mobile phone addiction, and they provide practical insights for enhancing students’ mental health and adaptive capabilities.

### 4.1 Grade-related differences in negative life events, perceived stress, psychological resilience, and mobile phone addiction

Grade functions as a differentiated background variable influencing psychological adaptation. This study identifies a gradient pattern: relative to their lower-grade peers, senior-year students report fewer negative life events, lower perceived stress, reduced tendencies toward mobile phone addiction, and higher levels of psychological resilience. This linear improvement trajectory partially supports the “Freshman Adaptation Stress Hypothesis,” which posits that first-year students experience peak perceived stress due to environmental transition and role reconfiguration (Liu and Li, 2024). However, this observed linear progression diverges from several findings in the extant literature. With regard to mobile phone dependence, prior studies frequently report a non-linear grade-based distribution: some indicate that dependence peaks among senior-year students (Zhang et al., 2020), while others find no statistically significant differences across grade levels (Fu et al., 2016). Regarding perceived stress, empirical evidence suggests a non-linear fluctuation pattern—first-year students typically confront the highest levels of environmental adaptation stress, whereas senior-year students often experience a marked rebound in stress attributable to employment pressures and graduation-related demands (Zhang et al., 2003). Similarly, concerning psychological resilience, research conducted with vocational college populations has revealed non-linear developmental trajectories; for instance, Guo (2013) found that second-year vocational college students exhibited significantly higher resilience than both first- and third-year counterparts.

The inconsistencies observed in this study relative to prior literature may stem from the distinctive demographic and educational characteristics of the study population. Specifically, three-year vocational college students undergo a condensed academic curriculum, and their progressive acquisition of professional competencies—coupled with pre-graduation internships—tends to accelerate the emergence of the “focus effect” on career goals. This early crystallization of vocational orientation likely strengthens senior students’ self-regulatory capacity and behavioral planning skills. With regard to psychological resilience, senior students’ accumulated experiences of successfully managing academic and occupational challenges may foster the incremental accumulation and consolidation of psychological resources over time. Regarding mobile phone addiction, the comparatively lower prevalence among senior students may be partly attributable to the increasing salience and prioritization of academic and career-related goals, which in turn facilitates adaptive reconfiguration of attentional allocation.

It is important to underscore that all interpretations presented above are grounded solely in statistical associations derived from cross-sectional data. Observed grade-level differences may reflect genuine developmental maturation, but they could also be confounded by cohort effects or selection biases. Longitudinal designs are therefore essential in future research to disentangle causal mechanisms underlying this grade-based gradient.

### 4.2 Direct association between negative life events and vocational college students’ mobile phone addiction

The results of this study suggest that the greater the number of negative life events experienced by vocational college students, the higher the likelihood of mobile phone addiction. In other words, negative life events are significantly and positively associated with mobile phone addiction behavior, consistent with Hypothesis 1. This finding aligns with previous studies and further supports the relevance of negative life events to the propensity for mobile phone addiction among this population (Wan, 2020; Wang, 2021; Zhou et al., 2022). It is widely recognized that transitioning from one educational stage to a higher one often entails significant changes in learning methods, living environments, and interpersonal relationships (Zhang and Peng, 2023). The theory of situational characteristics suggests that failure to adapt promptly to the evolving external environment may result in adverse outcomes such as mental health issues or difficulties in academic adaptation (Situ et al., 2021). In response to such challenges, individuals typically activate internal psychological adjustment mechanisms and employ strategies to mitigate the difficulties associated with life stressors. For example, they may use mobile phones to escape stress or regulate emotions (Yang et al., 2025). The interactive, recreational, and background management functions of mobile phones serve distinct roles in maintaining social connections, regulating emotions, and supporting learning, making them essential tools for coping with adverse life events. When individuals perceive improvements in their adaptive capacity in the context of mobile phone use, the frequency of this behavior increases alongside positive reinforcement and may eventually evolve into mobile phone addiction. These findings suggest that higher vocational colleges should pay close attention to the adverse effects of negative life events on students, assist them in managing negative emotions, guide them in reestablishing academic goals, and enhance their time management skills to reduce the risk of mobile phone addiction.

### 4.3 Indirect association between negative life events and vocational college students’ mobile phone addiction

#### 4.3.1 Mediating role of perceived stress

The research findings suggest that perceived stress serves as a mediating factor between negative life events encountered by vocational college students and their tendency toward mobile phone addiction. The data analysis is consistent with Hypothesis 2, demonstrating that the association of negative life events with mobile phone addiction is partially transmitted through increased perceived stress. Previous studies suggest that perceived stress is a psychological response that arises following life events and represents a key variable in the stress process (Brunnekreef et al., 2015). For vocational college students, adapting to new environments and managing academic and interpersonal pressures often result in heightened perceived stress stemming from negative life events, which in turn can influence their psychological and behavioral responses. The comprehensive model of stress processes highlights the dynamic interplay among environmental stressors, internal physiological and psychological regulatory mechanisms, and health outcomes (Ganzel et al., 2010). Prolonged exposure to stressful environments may disrupt an individual’s physical and mental equilibrium, potentially accompanied by psychological and behavioral issues such as social withdrawal and mobile phone addiction. Research has also shown that perceived stress is positively associated with mobile phone addiction; higher levels of perceived stress are associated with an increased likelihood of mobile phone dependency (Lian et al., 2018; Yin et al., 2022). Under stress, smartphones, due to their multifunctionality and social accessibility, can offer rapid psychological relief, allowing individuals to temporarily escape from stressors. With repeated use for emotional relief, individuals may gradually increase their mobile phone usage frequency, which may eventually co-occur with addictive behavior. These findings suggest that higher vocational colleges should focus on enhancing students’ stress coping strategies and emotional regulation abilities, as well as improving their academic focus under pressure, in order to prevent mobile phone addiction and promote academic success.

#### 4.3.2 The role of psychological resilience as a mediator

According to the findings of this study, psychological resilience serves as a mediating factor in the relationship between negative life events experienced by vocational college students and their tendencies toward mobile phone addiction. This conclusion is consistent with Hypothesis 3, which posits that negative life events are associated with lower psychological resilience, with a concomitant increase in the likelihood of mobile phone addiction. From the perspective of stress theory, negative life events function as psychosocial stressors that continuously deplete an individual’s psychological resources. When these resources are exhausted and compensatory mechanisms fail, individuals may struggle to manage environmental demands, potentially accompanied by maladaptive outcomes such as school aversion and mobile phone dependence (Hascher and Hagenauer, 2011). Previous research has shown that a greater frequency of negative life events correlates with a decline in psychological resilience (Masten, 2001; Zhang et al., 2021), suggesting that such events may indirectly be linked to challenges in adolescent adaptation and development by weakening this protective resource. Empirical evidence consistently demonstrates a significant negative correlation between psychological resilience and problematic mobile phone use. Higher levels of resilience are associated with a reduced risk of developing addictive behaviors related to mobile phone use (Dong et al., 2019; Ma and Zhou, 2024; Xu et al., 2025). Adolescents with high resilience are better able to mobilize positive emotions and cognitive resources when confronted with stressors such as academic pressure and interpersonal conflicts. They are more likely to employ problem-focused coping strategies and emotion regulation techniques, and exhibit lower reliance on mobile phones as an escape mechanism. In contrast, individuals with lower resilience are more prone to emotional exhaustion and feelings of helplessness and often resort to excessive mobile phone use as a short-term coping strategy, which may co-occur with heightened addictive behaviors. Therefore, higher vocational institutions can enhance students’ psychological resilience and academic engagement through classroom-based stress management training, academic support programs, and mentorship systems, ultimately reducing their dependency on mobile devices.

#### 4.3.3 Chain mediation of perceived stress and psychological resilience

The experimental data indicate that perceived stress and psychological resilience exhibit a chain-mediated effect between negative life events experienced by vocational college students and their tendency toward mobile phone addiction, consistent with Hypothesis 4. Specifically, negative life events initially elevate perceived stress levels among vocational college students, which are subsequently associated with diminished psychological resilience and a concomitant significant increase in mobile phone addiction tendencies. This finding aligns with previous research suggesting that different levels of perceived stress elicit different psychological resilience responses (Ye et al., 2018). Moreover, variations in psychological resilience can be linked to individuals’ emotional regulation and behavioral decisions under stress, with corresponding changes in the extent of their mobile phone dependence (Colomeischi and Ursu, 2024; Sun et al., 2025). First, negative life events exert a significant positive association with the perceived stress levels of vocational college students. An increase in the number of adverse experiences corresponds to heightened stress sensitivity and a more pronounced subjective stress response. Second, perceived stress demonstrates a significant negative association with psychological resilience; as stress levels rise, vocational college students exhibit diminished emotional regulation capacity and reduced ability to engage in adaptive coping strategies. Finally, psychological resilience is significantly and negatively correlated with mobile phone addiction: lower levels of resilience are linked to a higher likelihood of developing addictive behaviors. Consequently, perceived stress and psychological resilience jointly serve as a sequential mediating mechanism between negative life events and problematic mobile phone use among vocational college students. These findings suggest that negative life events not only are directly associated with mobile phone addiction but also exert an indirect association through the serial mediating effects of perceived stress and psychological resilience. The aforementioned chain mediation effect merely indicates that the observed data are statistically consistent with the hypothesized model. However, due to the cross-sectional design, the true temporal ordering among the variables cannot be established. Consequently, the proposed mediation pathway should be interpreted as a theoretically grounded and statistically supported explanatory framework—not as a causally validated sequence. Future longitudinal research will be essential to elucidate its directional dynamics.

### 4.4 Research implications and limitations

This study focuses on vocational college students to investigate the relationship and underlying psychological mechanisms between negative life events and mobile phone addiction. The findings indicate that negative life events are significantly associated with the occurrence of mobile phone addiction, with perceived stress and psychological resilience serving as independent and chained mediating factors in this relationship. By elucidating these psychological mechanisms, the study provides a theoretical foundation for potential interventions. It is recommended that higher vocational institutions and mental health professionals develop targeted support programs to help students manage stress and strengthen psychological resilience, thereby mitigating the risk of mobile phone addiction.

This study has several limitations. First, because of its cross-sectional design, causal relationships among the variables cannot be established. Future research may adopt longitudinal designs, such as cross-lagged panel models (CLPM), multilevel linear models (MLM), and latent growth models (LGM), to explore temporal and dynamic relationships among variables. Additionally, integrating qualitative methods, such as interviews and focus group discussions, could enhance the depth and richness of future investigations. Second, the inherent limitations of the cross-sectional design—coupled with reliance on self-reported data—preclude the complete elimination of confounding bias. Furthermore, regarding external validity, the sample was recruited via convenience sampling from only four colleges in a single province (Jiangxi), so caution is advised when generalizing the results to broader vocational populations. Therefore, future research should employ longitudinal designs or multi-source data collection strategies (e.g., supervisor–subordinate, peer–self, or self–other ratings) to further validate these findings. Third, this study only considered the mediating roles of perceived stress and psychological resilience in the relationship between negative life events and mobile phone addiction. Other potentially influential factors were not taken into account. Future studies could incorporate additional relevant variables to examine interactions among multilevel factors, thereby uncovering further potential moderating or mediating mechanisms and achieving a more comprehensive understanding of the underlying pathways.

## 5 Conclusion

In conclusion, this study revealed that negative life events, perceived stress, and psychological resilience are significantly correlated with mobile phone addiction. Moreover, perceived stress and psychological resilience exert both independent and sequential mediating effects in this relationship. These findings contribute to a deeper understanding of the psychological mechanisms underlying mobile phone addiction among vocational college students and expand the theoretical framework within adolescent mental health research. Given that the sample was recruited via convenience sampling from four vocational colleges in Jiangxi Province, the findings should be interpreted as most applicable to similar institutional and regional contexts. To establish broader generalizability, future research employing probability-based random sampling across multiple provinces is warranted. It is recommended that educational institutions implement stress management programs, foster a supportive campus environment, strengthen students’ psychological resilience, and offer services such as psychological counseling to assist students in coping with negative life events. When perceived stress is mitigated and psychological resilience is enhanced, mobile phone dependence can be effectively alleviated, which may contribute to students’ mental health and holistic development. It should be noted that, in this study, the term “addiction” adheres to the nomenclature of the adapted Chinese instrument (Xiong et al., 2012) and denotes patterns of problematic or excessive mobile phone use—not a clinically diagnosed behavioral addiction.

## Data Availability

The original contributions presented in the study are included in the article/Supplementary material, further inquiries can be directed to the corresponding author.
