# Supplementary material for: Negative life events and mobile phone addiction among Chinese vocational college students: a chain-mediation model of perceived stress and psychological resilience
Source: Front Psychol. 2026 Jun 17;17:1854552. doi: 10.3389/fpsyg.2026.1854552 (PMC13318680; doi:10.3389/fpsyg.2026.1854552)
Supplement: Supplementary file 2 [file Table_2.DOCX]

Supplementary Material

**Negative Life Events and Mobile Phone Addiction among Vocational College Students: a Chain-Mediation Model of Perceived Stress and Psychological Resilience**

Lijuan Xu^1^, Li Li^2*^

*** Correspondence:** Li Li: [lancylili@ncu.edu.cn](mailto:lancylili@ncu.edu.cn)

**1 Introduction**

Dear classmates, please help me with my academic research by completing this questionnaire. Your answers will provide me with valuable research data. This questionnaire is anonymous, and there is no right or wrong answer—instead, please choose the ones that best suit you. Thank you for participating in this survey!

The questionnaire consists of four parts composed of 27, 14, 10 and 16 questions, respectively. The questionnaire should take 5 min to complete. Thank you again for your attention.

**2 Basic Information**

1. Your gender: ① Male ② female
2. Your grade: ① First year of higher Vocational Education

② Second year of higher Vocational Education

③ Third year of higher Vocational Education

3. Originating Place: ① Urban (county, city, province) ② Rural (township, town, village)

**3** Adolescent Self-Rating Life Events Scale **( D )**

Please read the following items carefully and select the one that best aligns with your preferences.

**d1** 4. Be misunderstood or wrongly accused by others.

①No occurrence/No impact ② Moderate impact ③ Mild impact

④ Severe impact ⑤ Extremely severe impact

**d2** 5. Discrimination or cold treatment by others.

①No occurrence/No impact ② Moderate impact ③ Mild impact

④ Severe impact ⑤ Extremely severe impact

**d3**  6. Failure or unsatisfactory performance in the exam.

①No occurrence/No impact ② Moderate impact ③ Mild impact

④ Severe impact ⑤ Extremely severe impact

**d4** 7. Have disputes with classmates or close friends.

①No occurrence/No impact ② Moderate impact ③ Mild impact

④ Severe impact ⑤ Extremely severe impact

**d5** 8. There are obvious changes in living habits (such as diet and rest).

①No occurrence/No impact ② Moderate impact ③ Mild impact

④ Severe impact ⑤ Extremely severe impact

**d6** 9. Don't like going to school.

①No occurrence/No impact ② Moderate impact ③ Mild impact

④ Severe impact ⑤ Extremely severe impact

**D7** 10. An unsuccessful relationship or a broken heart.

①No occurrence/No impact ② Moderate impact ③ Mild impact

④ Severe impact ⑤ Extremely severe impact

**d8** 11. Being away from family for a long time and unable to reunite.

①No occurrence/No impact ② Moderate impact ③ Mild impact

④ Severe impact ⑤ Extremely severe impact

**d9** 12. Heavy study burden.

①No occurrence/No impact ② Moderate impact ③ Mild impact

④ Severe impact ⑤ Extremely severe impact

**d10** 13. Tense relationship with the teacher.

①No occurrence/No impact ② Moderate impact ③ Mild impact

④ Severe impact ⑤ Extremely severe impact

**d11** 14. I am suffering from a serious and acute illness.

①No occurrence/No impact ② Moderate impact ③ Mild impact

④ Severe impact ⑤ Extremely severe impact

**d12** 15. A relative or friend is suffering from a serious or acute illness.

①No occurrence/No impact ② Moderate impact ③ Mild impact

④ Severe impact ⑤ Extremely severe impact

**d13** 16. Death of a relative or friend.

①No occurrence/No impact ② Moderate impact ③ Mild impact

④ Severe impact ⑤ Extremely severe impact

**d14** 17. Theft or loss of Dongyou.

①No occurrence/No impact ② Moderate impact ③ Mild impact

④ Severe impact ⑤ Extremely severe impact

**d15** 18. Losing face in public

①No occurrence/No impact ② Moderate impact ③ Mild impact

④ Severe impact ⑤ Extremely severe impact

**d16** 19. The family is in financial difficulty.

①No occurrence/No impact ② Moderate impact ③ Mild impact

④ Severe impact ⑤ Extremely severe impact

**d17** 20. There are conflicts within the family.

①No occurrence/No impact ② Moderate impact ③ Mild impact

④ Severe impact ⑤ Extremely severe impact

**d18** 21. The expected selection (such as the Title of "Outstanding Student") failed

①No occurrence/No impact ② Moderate impact ③ Mild impact

④ Severe impact ⑤ Extremely severe impact

**d19** 22. Be criticized or punished.

①No occurrence/No impact ② Moderate impact ③ Mild impact

④ Severe impact ⑤ Extremely severe impact

**d20** 23. Transfer or withdrawal from school.

①No occurrence/No impact ② Moderate impact ③ Mild impact

④ Severe impact ⑤ Extremely severe impact

**d21** 24. Be punished.

①No occurrence/No impact ② Moderate impact ③ Mild impact

④ Severe impact ⑤ Extremely severe impact

**d22** 25. Pressure of further education.

①No occurrence/No impact ② Moderate impact ③ Mild impact

④ Severe impact ⑤ Extremely severe impact

**d23** 26. Fighting with others.

①No occurrence/No impact ② Moderate impact ③ Mild impact

④ Severe impact ⑤ Extremely severe impact

**d24** 27. Be scolded by parents.

①No occurrence/No impact ② Moderate impact ③ Mild impact

④ Severe impact ⑤ Extremely severe impact

**d25** 28. Your family puts pressure on you to study.

①No occurrence/No impact ② Moderate impact ③ Mild impact

④ Severe impact ⑤ Extremely severe impact

**d26** 29. Unexpected shock, accident.

①No occurrence/No impact ② Moderate impact ③ Mild impact

④ Severe impact ⑤ Extremely severe impact

**d27** 30. Others.

①No occurrence/No impact ② Moderate impact ③ Mild impact

④ Severe impact ⑤ Extremely severe impact

**4** Perceived Stress Scale **( E )**

This questionnaire is designed to assess your perceived level of stress over the past month. Please place a checkmark ("√") next to the option that best reflects your experience.

**e1** 31. Feel upset about the occurrence of some unexpected things.

① Completely inconsistent ② Relatively inconsistent ③ Uncertain

④ relatively consistent ⑤ Completely consistent

**e2** 32. I feel unable to control the important things in my life.

① Completely inconsistent ② Relatively inconsistent ③ Uncertain

④ relatively consistent ⑤ Completely consistent

**e3** 33. Feel nervous, uneasy and stressed

① Completely inconsistent ② Relatively inconsistent ③ Uncertain

④ relatively consistent ⑤ Completely consistent

**e4** 34. Successfully handle the annoying things in life.

① Completely inconsistent ② Relatively inconsistent ③ Uncertain

④ relatively consistent ⑤ Completely consistent

**e5** 35. Feel that one can effectively handle the important changes that occur in life.

① Completely inconsistent ② Relatively inconsistent ③ Uncertain

④ relatively consistent ⑤ Completely consistent

**e6** 36. Feel confident in having the ability to handle one's own personal issues.

① Completely inconsistent ② Relatively inconsistent ③ Uncertain

④ relatively consistent ⑤ Completely consistent

**e7**  37. Feeling that things are developing as one wishes.

① Completely inconsistent ② Relatively inconsistent ③ Uncertain

④ relatively consistent ⑤ Completely consistent

**e8** 38. I feel that I can't handle all the things I have to do.

① Completely inconsistent ② Relatively inconsistent ③ Uncertain

④ relatively consistent ⑤ Completely consistent

**e9** 39. Be able to solve unpleasant things in life.

① Completely inconsistent ② Relatively inconsistent ③ Uncertain

④ relatively consistent ⑤ Completely consistent

**e10** 40. I often feel that I am the master of everything.

① Completely inconsistent ② Relatively inconsistent ③ Uncertain

④ relatively consistent ⑤ Completely consistent

**e11** 41. Be angry that something has happened beyond one's control.

① Completely inconsistent ② Relatively inconsistent ③ Uncertain

④ relatively consistent ⑤ Completely consistent

**e12** 42. I find myself thinking about some things that must be done.

① Completely inconsistent ② Relatively inconsistent ③ Uncertain

④ relatively consistent ⑤ Completely consistent

**e13** 43. Always be able to master the way of arranging time.

① Completely inconsistent ② Relatively inconsistent ③ Uncertain

④ relatively consistent ⑤ Completely consistent

**e14** 44. Feeling that the problem is constantly accumulating and cannot be solved.

① Completely inconsistent ② Relatively inconsistent ③ Uncertain

④ relatively consistent ⑤ Completely consistent

**5** Psychological Resilience Scale **( F )**

Please choose the option that best suits your feelings based on your situation in the past month to answer.

**f1** 45. When things change, I can adapt.

① Never ② Rarely ③ Sometimes ④ Frequently ⑤ always

**f2** 46. No matter what happens on the journey of life, I can handle it.

① Never ② Rarely ③ Sometimes ④ Frequently ⑤ always

**f3** 47.When facing difficult problems, I try to see the positive side of things.

① Never ② Rarely ③ Sometimes ④ Frequently ⑤ always

**f4** 48. Going through hardships will make me stronger.

① Never ② Rarely ③ Sometimes ④ Frequently ⑤ always

**f5** 49. I recover easily from illness, injury or difficulty.

① Never ② Rarely ③ Sometimes ④ Frequently ⑤ always

**f6** 50. I believe that even if I encounter obstacles, I can still achieve my goals.

① Never ② Rarely ③ Sometimes ④ Frequently ⑤ always

**f7** 51. Under pressure, I can still concentrate on thinking about problems.

① Never ② Rarely ③ Sometimes ④ Frequently ⑤ always

**f8** 52. I won't be easily defeated by failure.

① Never ② Rarely ③ Sometimes ④ Frequently ⑤ always

**f9** 53. When dealing with failures and difficulties in life, I think I am a strong person.

① Never ② Rarely ③ Sometimes ④ Frequently ⑤ always

**f10** 54. I can handle some unpleasant or painful feelings, such as sadness, fear and anger.

① Never ② Rarely ③ Sometimes ④ Frequently ⑤ always

**6** Mobile Phone Addiction Questionnaire **( G )**

For each of the following items, please evaluate it based on your actual situation.

**g1** 55. If I haven't brought my phone for a while, I will immediately check if there are any text messages or missed calls.

①Very inconsistent ② Not quite consistent ③ Average

④ relatively consistent ⑤ Very consistent

**g2** 56. I would rather chat on my mobile phone than communicate face to face directly.

①Very inconsistent ② Not quite consistent ③ Average

④ relatively consistent ⑤ Very consistent

**g3** 57. When waiting for someone, I always call my phone frequently to ask where they are. If they don't call, I get extremely anxious.

①Very inconsistent ② Not quite consistent ③ Average

④ relatively consistent ⑤ Very consistent

**g4** 58. If I don't use my mobile phone for a long time, I will feel uncomfortable

①Very inconsistent ② Not quite consistent ③ Average

④ relatively consistent ⑤ Very consistent

**g5** 59. In class, I can't concentrate because of phone calls or text messages.

①Very inconsistent ② Not quite consistent ③ Average

④ relatively consistent ⑤ Very consistent

**g6** 60. I would feel lonely without my mobile phone.

①Very inconsistent ② Not quite consistent ③ Average

④ relatively consistent ⑤ Very consistent

**g7** 61.When communicating with others on my mobile phone, I feel more confident.

①Very inconsistent ② Not quite consistent ③ Average

④ relatively consistent ⑤ Very consistent

**g8** 62. If my phone doesn't ring for a while, I will feel uncomfortable and subconsciously check if there are any missed calls or text messages on it.

①Very inconsistent ② Not quite consistent ③ Average

④ relatively consistent ⑤ Very consistent

**g9** 63. I often have the illusion that my phone is ringing/my phone is vibrating.

①Very inconsistent ② Not quite consistent ③ Average

④ relatively consistent ⑤ Very consistent

**g10** 64. If I have more phone calls and text messages, I will feel that my life is more fulfilling.

①Very inconsistent ② Not quite consistent ③ Average

④ relatively consistent ⑤ Very consistent

**g11** 65. I'm often afraid that my mobile phone will shut down automatically.

①Very inconsistent ② Not quite consistent ③ Average

④ relatively consistent ⑤ Very consistent

**g12** 66. My mobile phone is a part of me. Once I lose it, I feel as if I have lost something.

①Very inconsistent ② Not quite consistent ③ Average

④ relatively consistent ⑤ Very consistent

**g13** 67.My classmates and friends often say that I am too dependent on my mobile phone.

①Very inconsistent ② Not quite consistent ③ Average

④ relatively consistent ⑤ Very consistent

**g14** 68. When my phone often fails to connect or receive a signal, I get anxious and my temper becomes irritable.

①Very inconsistent ② Not quite consistent ③ Average

④ relatively consistent ⑤ Very consistent

**g15** 69.In class, I often take the initiative to focus my attention on my mobile phone, which affects my listening.

①Very inconsistent ② Not quite consistent ③ Average

④ relatively consistent ⑤ Very consistent

**g16** 70. I think it's more comfortable to communicate with others via mobile phones.

①Very inconsistent ② Not quite consistent ③ Average

④ relatively consistent ⑤ Very consistent

Note: D = Adolescent Self-Rating Life Events Scale ( Negative life events )

E = Perceived Stress Scale ( Perceived stress )

F = Psychological Resilience Scale ( Psychological resilience )

G = Mobile Phone Addiction Questionnaire ( Mobile phone addiction )
